# *Drosophila* ataxin‐2 gene encodes two differentially expressed isoforms and its function in larval fat body is crucial for development of peripheral tissues

**DOI:** 10.1002/2211-5463.12124

**Published:** 2016-10-07

**Authors:** Murilo Carlos Bizam Vianna, Deise Cristina Poleto, Paula Fernanda Gomes, Valéria Valente, Maria Luisa Paçó‐Larson

**Affiliations:** ^1^ Department of Cellular and Molecular BiologyRibeirão Preto School of MedicineUniversity of São PauloRibeirão PretoSPBrazil; ^2^ Present address: Center of Biological SciencesState University of LondrinaCampus Universitário, LondrinaPR 86057‐97Brazil; ^3^ Present address: Department of Clinical AnalysisFaculty of Pharmaceutical Sciences of AraraquaraUniversity of São Paulo State (UNESP)R. Expedicionários do Brasil, 1628, AraraquaraSP 14801‐902Brazil

**Keywords:** dAtx2 knockdown, developmental regulation, Drosophila ataxin‐2 isoforms, larval fat body, subcellular localization

## Abstract

Different isoforms of ataxin‐2 are predicted in Drosophila and may underlie different cellular processes. Here, we validated the isoforms B and C of Drosophila ataxin‐2 locus (*dAtx2*), which we found to be expressed in various tissues and at different levels during development. dAtx2‐B mRNA was detected at low amounts during all developmental stages, whereas dAtx2‐C mRNA levels increase by eightfold from L3 to pupal–adult stages. Higher amounts of dAtx2‐B protein were detected in embryos, while dAtx2‐C protein was also expressed in higher levels in pupal–adult stages, indicating post‐transcriptional control for isoform B and transcription induction for isoform C, respectively. Moreover, in the fat body of L3 larvae dAtx2‐C, but not dAtx2‐B, accumulates in cytoplasmic foci that colocalize with sec23, a marker of endoplasmic reticulum exit sites (ERES). Interestingly, animals subjected to selective knockdown of dAtx2 in the larval fat body do not complete metamorphosis and die at the third larval stage or early puparium. Additionally, larvae knocked down for dAtx2, grown at 29 °C, are significantly smaller than control animals due to reduction in DNA replication and cell growth, which are consistent with the decreased levels of phosphorylated‐AKT observed in the fat body. Based on the localization of ataxin‐2 (dAtx2‐C) in ERESs, and on the phenotypes observed by dAtx2 knockdown in the larval fat body, we speculate a possible role for this protein in processes that regulate ERES formation. These data provide new insights into the biological function of ataxin‐2 with potential relevance to neurodegenerative diseases.

**Abbreviations**
AKT: PKB (protein kinase B)
dAtx2‐B: isoform B of *Drosophila* ataxin‐2
dAtx2‐C: isoform C of *Drosophila* ataxin‐2
dsAtx2: double‐stranded RNA for Drosophila ataxin‐2
ERES: endoplasmic reticulum exit site
PI3K: phosphatidylinositol 3‐kinase
rER: endoplasmic reticulum
α‐dAtx2 B + C: antibody that detects both isoforms B and C
α‐dAtx2 B: antibody specific for isoform B

*Drosophila dAtx2* is the ortholog of the human gene ATXN2 (Satterfield *et al*., 2002). ATXN2 is associated with the spinocerebellar ataxia type‐2 (SCA2), which belongs to the family of the so‐called polyglutamine diseases that include various ataxias among other neurodegenerative disorders 1. Despite the lack of a clear relationship between the ataxia‐causing genes, many of their products interact physically and genetically suggesting that the SCAs, and perhaps other ataxias, share common mechanisms of pathogenesis 2, 3, 4, 5, 6. Also, more recent reports showed the interaction of ataxin‐2 with TDP43 7, 8, a RNA‐binding protein associated with the amyotrophic lateral sclerosis (ALS). Moreover, a survey of modifier genes in *Saccharomyces cerevisiae*,* Caenorhabditis elegans,* and *Drosophila melanogaster* concluded that ataxin‐2 orthologs are generic modifiers that affect multiple if not all neurodegenerative diseases 9, reinforcing the relevance of investigations on the roles of wild‐type ataxin‐2.

Analysis of Atxn2^−/−^ mice revealed an increase of several global translation factors in their transcript abundance, but decreases in the translation rate 10. Indeed, studies on the members of ataxin‐2 in different organisms have implicated these proteins in post‐transcription regulation. Ataxin‐2 physically interacts with poly (A)‐binding protein (PABP) 11, 12, 13. Besides cosedimentation with PABP in polysomes 13, ataxin‐2 and Pbp1 (yeast ortholog of ataxin‐2) are implicated in the assembly of stress granules, a ribonucleic acid protein complex engaged in stress‐triggered translational arrest 14, in both mammalian cells and yeast 12, 15, 16. Recent *in vivo* studies show that dAtx2 also is required for microRNA function 17, coordinates an active translation complex important for PER expression in circadian neurons 18, 19 and functions in long‐term memory, regulating translation of presynaptic and postsynaptic mRNA 20. It was also shown in human cells that ataxin‐2 directly binds 3′ UTRs elements promoting mRNA stabilization and protein expression 21.

Ataxin‐2 has also been associated with processes other than post‐transcription regulation. It has been shown to bind to endophilin A1/3 in mammalian cells, with a possible role in endocytic receptor cycling 22 and to be involved in cell signaling 23. It was also reported to bind the transcription factor ZBRK1, possibly acting as a coactivator of its own transcription 24.

ATXN2 expresses alternative spliced isoforms, which are conserved in mouse 25, 26. These isoforms are present in several human tissues including brain, spinal cord, cerebellum, heart, and placenta. In the central nervous system, the larger ataxin‐2 mRNA predominates in the brain and spinal cord, while the splice variant II is more abundant in the cerebellum 25. Northern blot analysis of different mouse tissues also indicated that the mouse ataxin‐2 isoforms are expressed in most tissues, but at varying levels 27. As is for the mammalian ortholog, it is predicted that the Drosophila *dAtx2* gene expresses three mRNA encoding different ORFs, named dAtx2‐A, ‐B, and ‐C (Fig. 1A). Since ataxin‐2 isoforms may underlie different cellular processes, their further characterization is still necessary.

**Figure 1:**
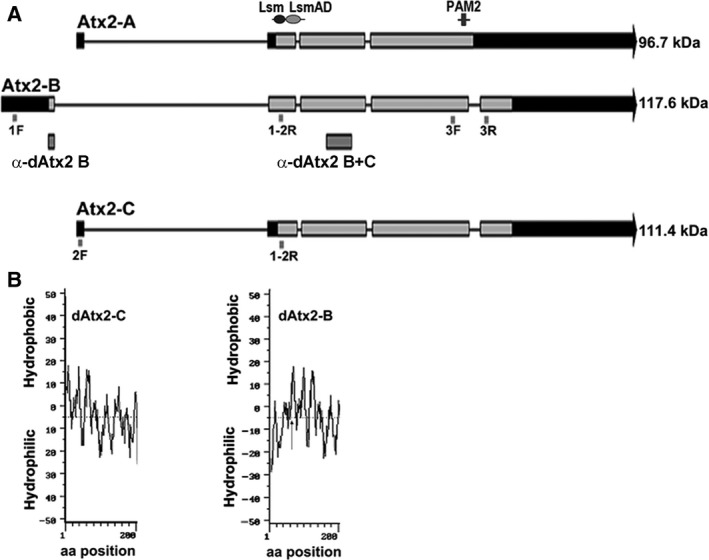
Structure of the *dAtx2* predicted isoforms. (A) Schematic representation of the exon–intron organization of *Drosophila melanogaster* predicted isoforms: dAtx2‐A, dAtx2‐B, and dAtx2‐C. The exons are indicated by bars and the introns by lines. The arrowheads indicate the transcription direction. Noncoding sequences are shown as black portions of the exon bars. The localization of the primer sequences used in the mRNA expression analysis is indicated. The conserved Lsm/LsmAD domains and PAM‐2 motif are indicated. The positions of the fragments expressed in bacteria for the generation of antibodies (α‐dAtx2 B and α‐dAtx2 B + C) and the estimated mass of the protein isoforms are indicated. (B) The index of hydropacity of the ataxin‐2 isoforms A and B amino‐terminal (200 amino acids). The extra 61 residues in the N terminus, specific of dAtx2‐B (arrow), confer a hydrophilic character to the amino‐terminal region of this isoform.

In the present work we validate the existence of the isoforms B and C of *Drosophila* ataxin‐2, which are shown to be differentially expressed during development at the mRNA and protein levels. Interestingly, by RNA interference‐mediated depletion of dAtx2 we show that limiting levels of ataxin‐2 in the larval fat body are required for proper development of the organism. Furthermore, the localization of ataxin‐2 (dAtx2‐C) in the ERESs of fat body cells suggest a possible role of this protein in the ERES function, which could explain the phenotypes resulting from depletion of dAtx2 in this tissue.

## Results

### Analysis of the expression of ataxin‐2 in different tissues and developmental stages

**Figure 2:**
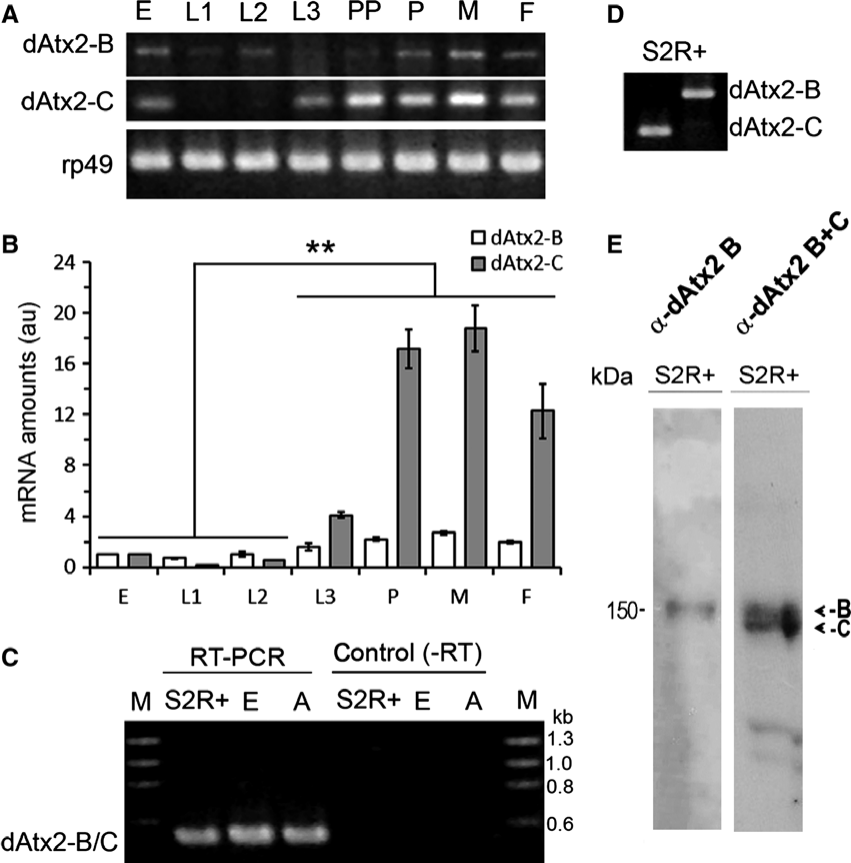
Validation of dAtx2‐B and dAtx2‐C isoforms expression and quantification of the mRNA levels during development. (A) total RNA extracted from embryo (E), larvae at first (L1), second (L2), and third (L3) instar, prepupae (PP), pupa (P), and adult male (M) or female (F) and S2R+ cells (C, D) was analyzed by RT/PCR using pairs of primers directed to the first exons of isoform B (1F) or isoform C (2F), and to the second exon (1/2R) shared by both isoforms (see Fig. 1). (B) The levels of transcript dAtx2‐C significantly increased from the third larval stage. The graph shows the relative quantification of mRNAs dAtx2‐B (white) and dAtx2‐C (gray) during the stages of embryo (E); larva first (L1) second (L2) and third stage (L3); pupa (P); male (M) and female (F) RT‐qPCR. The average values (±SD) correspond to the normalized amounts of dAtx2‐B and ‐C in arbitrary units; *n* = 3, **P < 0.01. (C) Total RNA extracted from S2R+ cells, embryo (E) and adult flies was submitted to RT/PCR analysis using a pair of primers directed to the exons flanking the last intron (3F and 3R, see Fig. 1A). Duplicates in which the reverse transcriptase was omitted (‐RT) were used as controls. (E) Western blots of S2R+ cells protein extract probed with α‐dAtx2 B (specific for isoform B) or α‐dAtx2 B + C (detects both isoforms B and C), developed by chemoluminescence (ECL).

A cDNA fragment common to the dAtx2 mRNA predicted in the flybase databank detected only one band of about 5.6 kb in northern blot (result not shown). Thus, to validate the existence of dAtx2‐A, ‐B, and ‐C mRNA, primer‐specific RT‐PCR was performed (see Methods and Fig. 1A). The analysis of RNA extracted from animals at different developmental stages revealed the presence of dAtx2‐B at all stages, whereas in postembryonic stages, dAtx2‐C mRNA was detected only after L3 (Fig. 2A). Indeed, by qRT‐PCR we observed that the levels of dAtx2‐B remain relatively low throughout development, while the levels of dAtx2‐C increased about eight times from L3 to pupal/adult stages (*n* = 3, *P* < 0.01) (Fig. 2B). Both isoforms, dAtx2‐B and ‐C, were also detected in RNA extracted from S2R+ cells (Fig. 2D). RT‐PCR using primers directed to the exons flanking the fourth intron, which is retained in the dAtx2‐A isoform (Fig. 1A), did not detect the expected dAtx2‐A fragment in any of the analyzed tissues, S2R+ cells, embryos or adult flies (Fig. 2C).

The first methionine that defines the dAtx2‐B ORF is in frame with the first methionine of the dAtx2‐C ORF raising the possibility that both transcripts express the same polypeptide. If dAtx2‐B translation initiates in the first methionine, the expressed polypeptide would contain an additional 61 amino acid residues exclusive of this isoform. The presence of this additional peptide in dAtx2‐B would change the hydropathical character of the N terminus from hydrophobic to hydrophilic (Fig. 1B), which could have potential functional significance. Therefore, to search for dAtx2‐B, a polyclonal antibody named α‐dAtx2 B was generated against the 61 amino acid fragment specific of this isoform. As expected, the affinity purified α‐dAtx2 B was able to detect a unique band while α‐dAtx2 B + C detected two bands of similar SDS/PAGE mobility (~ 150 kDa) in samples of S2R+ cell extract treated with 8 m urea in 6% SDS/PAGE (Fig. 2E). The signal detected by both antibodies in samples of tissues silenced for ataxin‐2 are significantly reduced compared to control (Figs 4, 8, and 9), confirming the antibody specificity.

**Figure 3:**
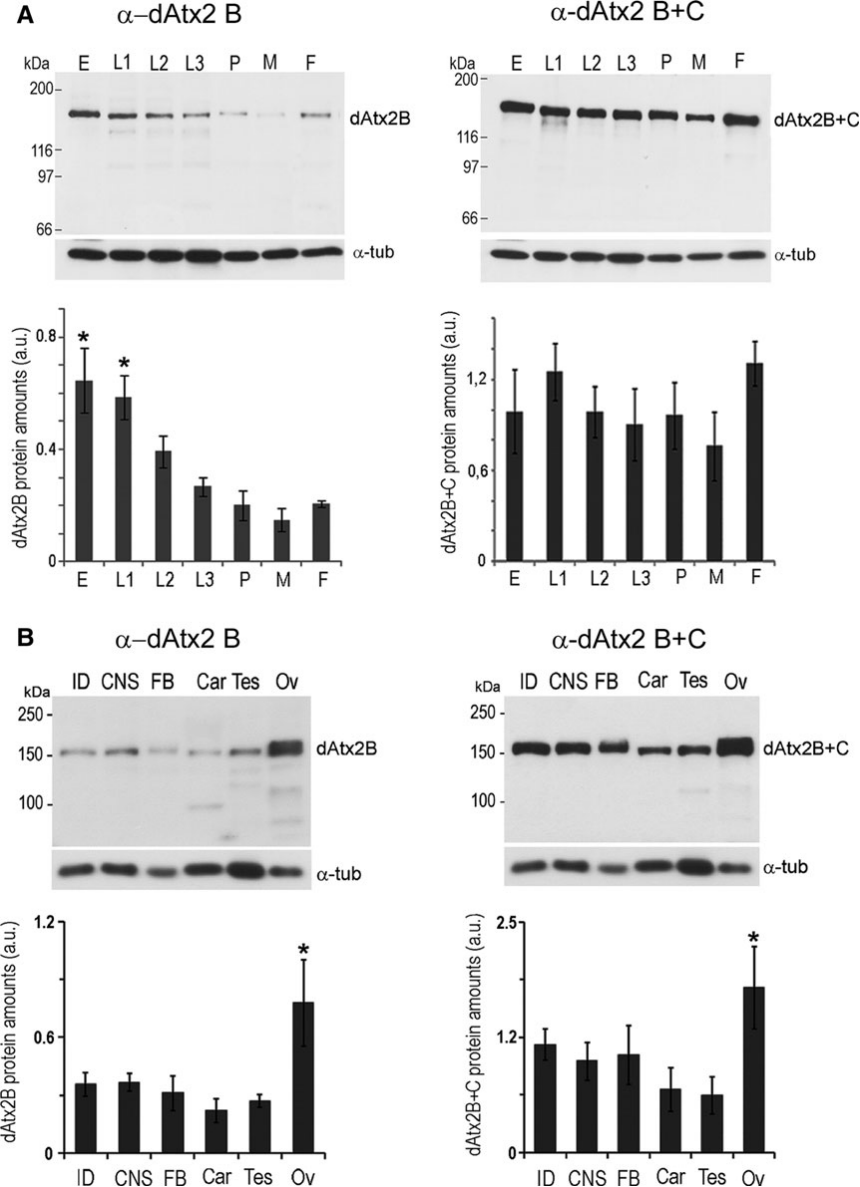
Expression pattern of dAtx2 during development and in different tissues. (A) protein extracts of embryo (E), larva at first (L1), second (L2) and third stage (L3), pupa and adults female (F) and male (M). (B) protein extracts of dissected tissues: imaginal disks (ID), central nervous system (CNS), fat body (FB), and carcass (Car) of third instar larvae, and testes and ovary (OV) of adult flies. The western blots were probed with α‐dAtx2 B or α‐dAtx2 B + C, and α‐tubulin for loading normalization. Densitometry analysis of the bands corresponding to dAtx2‐B or dAtx2‐B plus dAtx2‐C, and antiα‐tubulin proteins were performed with imagej (http://rsbweb.nih.gov/ij/). The values are means (±SD) of the pixel intensities corresponding to the normalized amounts of dAtx2 expressed in arbitrary units (a.u.); *n* = 3. Analysis of variance (one‐way ANOVA) was performed for data and significance is indicated by an asterisk; *P < 0.05.

To analyze the protein levels of dAtx2 isoforms, the signals of both antibodies (α‐dAtx2 B and α‐dAtx2 B + C) were quantified in western blots of protein extracts of whole animals or dissected tissues. We found that the amounts of dAtx2‐B isoform are significantly higher in embryos and L1 larvae (*n* = 3, *P* < 0.05) than in larvae at stages L2–L3, pupa, and adults. Differently, α‐dAtx2 B + C signal intensity was similar in all stages analyzed (Fig. 3A). The analysis of protein extracts of larval (imaginal disks, central nervous system, and fat body) and adult (testis and ovary) tissues showed that both antibody signals are significantly higher (*n* = 3, *P* < 0.05) in ovaries compared to the other tissues analyzed (Fig. 3B). Together, these data indicate that ataxin‐2 isoforms are present in different quantities during development, and both proteins are present in higher amounts in the ovary of adult compared to the other tissues.

### The effect on growth and development of dAtx2 knockdown in the fat body

**Figure 4:**
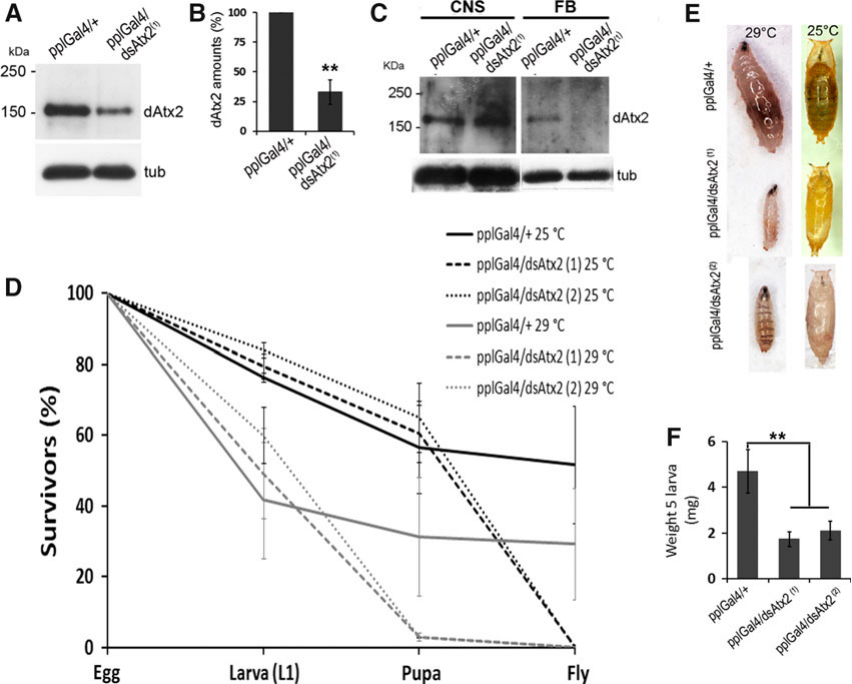
The effect of RNA interference mediated reduction of ataxin‐2 levels in the fat body on growth and survival of *Drosophila*. (A) Western blot of protein extracts from fat body of larvae expressing dsRNA against dAtx2 in fat body (pplGal4/dsAtx2^(1)^) and control (pplGal4/+), probed with α‐dAtx2 B + C. (B) The graph represents the densitometry of the dAtx2 band normalized against α‐tubulin band, *n* = 3, **P < 0.01. (C) Western blot of protein extracts of central nervous system (CNS) and fat body (FB) larvae expressing dsRNA against dAtx2 in fat body (pplGal4/dsAtx2^(1)^) and control (pplGal4/+), probed with α‐dAtx2 B + C. (D) Graph of percentage of survival (mean ± SEM), control (*n* = 1164), and silenced groups (dsAtx2^(1)^, *n* = 1374; dsAtx2^(2)^, *n* = 803) maintained at 25 °C; control (*n* = 710) and silenced groups (dsAtx2^(1)^, *n* = 1009; dsAtx2^(2)^, *n* = 577) at 29 °C. (E) Image of 3rd stage larvae (96 h after egg deposition, AED) grown at 29 °C of ataxin‐2 silenced (pplGal4/dsAtx2^(1)^ and/dsAtx2^(2)^) and control (pplGal4/+) groups. The images were obtained using Leica stereoscope equipped with digital camera. (F) The graph represents average ±SEM for each group. (*n* = 50, **P< 0.01).

Considering that ataxin‐2 knockout mice present reductions in the insulin receptor levels 23 and that PI3K activity in the fat body of *Drosophila* is required for organism and cellular growth 28, 29 we examined the effect of dAtx2 knockdown in the larval fat body on *Drosophila* development. For this, antisense transgene directed against dAtx2 was expressed in the fat body (ppl‐GAL4 driving UAS‐dsdAtx2; hereafter called pplGal4/dsAtx2). As expected, the levels of ataxin‐2 in the fat body of pplGal4/dsAtx2^(1)^ larvae were ~ 65% lower than the control pplGal4/+ (Fig. 4A,B) and were not affected in the CNS, for instance (Fig. 4C). At 25 °C, the larvae with reduced levels of ataxin‐2 in the fat body did not complete metamorphosis and died in the pupal case (Fig. 4D). Although the larval cuticle became the pupal case, the formation of adult structures such as eyes, wings, and legs was not observed in the dAtx2 knockdown animals (Fig. 4E). At 29 °C, the phenotype was even more severe. The rate of survival of pplGal4/dsAtx2^(1)^ animals at L3 was significantly reduced, and only 2.8% of these larvae turned into prepupa (Fig. 4D). Moreover, we observed that at L3 (96 h after egg deposition) pplGal4/dsAtx2^(1)^ larvae grown at 29 °C were smaller and 2.5‐fold lighter than the control larvae (Fig. 4E,F). Similar phenotype was obtained using one additional VDRC RNAi line (UAS‐dsdAtx2^(2)^, Fig. 4E,F), which specificity toward ataxin‐2 was previously validated 17, 20.

**Figure 5:**
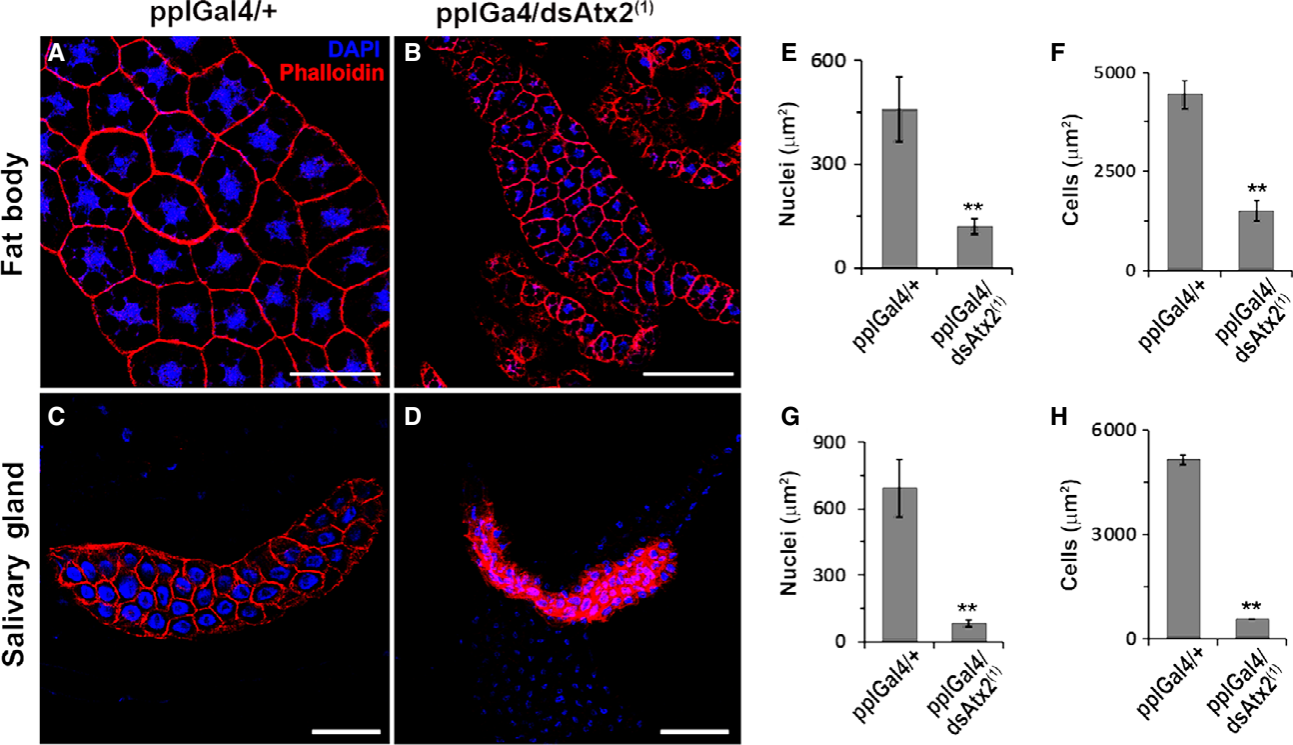
Ataxin‐2 knockdown in the fat body causes reduction of the cell size in the larval fat body and salivary gland. (A–D) Fat body and salivary glands of pplGal4/dsAtx2^(1)^ and pplGal4/+ 3rd larvae (96 h AED) were dissected and the actin cortex labeled with phalloidin conjugated to Alexa Fluor 594 (Molecular Probes, Waltham, MA, USA) and the nuclei stained with 4′,6‐diamidino‐2‐phenylindole, fluorescent stain that binds strongly to A‐T rich regions in DNA. Images were captured using confocal microscope Leica TCS SP5. (E, F) The graphs represent the average ±SEM of the area of cells (*n* = 30) and nuclei (*n* = 40) of fat body and salivary glands of pplGal4/dsAtx2^(1)^ e pplGal4/+. Significance levels based on analysis of variance (one‐way ANOVA) are indicated: **P < 0.01. Scale bars = 100 μm.

**Figure 6:**
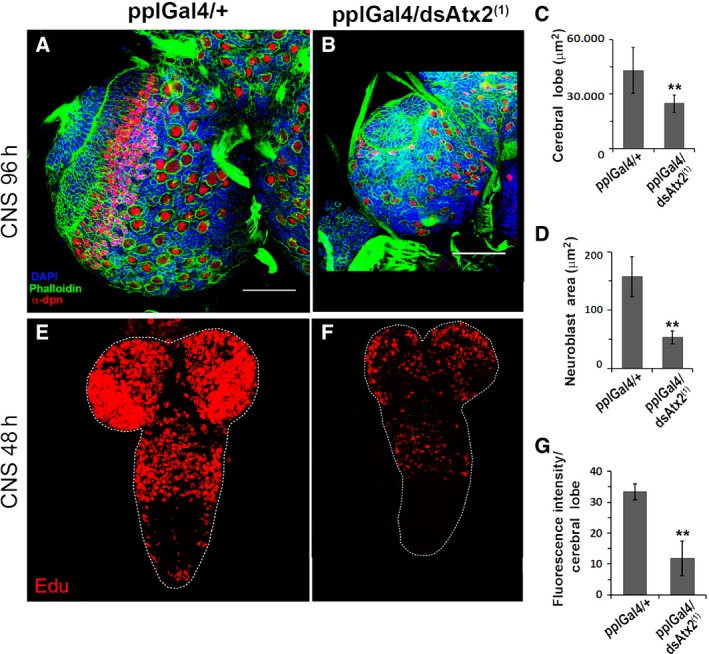
Ataxin‐2 knockdown in the fat body decreases the size of the cerebral lobes, the area of neuroblasts, and reduces but does not arrest replication in the CNS. (A, B) Brain lobes of third‐stage larvae (96 h AED) of pplGal4/dsAtx2^(1)^ and pplGal4/+ were dissected and the neuroblasts were immunostained with α‐dpn and α‐mouse IgG conjugated to Texas Red, actin filaments were stained with phalloidin conjugated to Alexa Fluor 488 and nuclei with 4′,6‐diamidino‐2‐phenylindole, fluorescent stain that binds strongly to A‐T rich regions in DNA. The images were captured using the Leica TCS SP5 confocal microscope (A, B). Scale bars = 100 μm. The graphs represent the mean ± SEM (area of the cerebral lobes: *n* = 4; area of neuroblasts: *n* = 52, **P < 0.01) areas of the cerebral lobes (C) and neuroblasts (D) of pplGal4/dsAtx2^(1)^ and pplGal4/+. (E, F) CNS second‐stage larvae (48 h AED) pplGal4/dsAtx2^(1)^ and pplGal4/+ were dissected, followed by incorporation with EdU (5‐ethynyl‐2′‐deoxyuridine) and covalently binding to Alexa Fluor 594. (G) Graph resulting from the sum of the values of pixel intensity normalized by the cerebral lobes areas (*n* = 4, **P < 0.01).

To verify if the reduced growth of pplGal4/dsAtx2 animals maintained at 29 °C is a consequence of reduced cell sizes or replication indexes, or both, larval tissues that were grown by endoreplication or mitotic proliferation were analyzed by morphometry and/or deoxyribonucleotide incorporation. The results showed that the cells of the fat body (Fig. 5A,B) and salivary gland (Fig. 5C, D) of pplGal4/dsAtx2^(1)^ larvae had an average area 2.9 times and 9.1 times, respectively, smaller than the controls (Fig. 5F,H). In addition, the area of fat body and salivary gland nuclei were respectively 3.8 times and 8.6 times smaller compared to controls (Fig. 5E,G). For analysis of a tissue that grows by mitotic proliferation we used central nervous system (CNS). At 29 °C, the cerebral lobe and neuroblasts of pplGal4/dsAtx2^(1)^ larvae presented an area 3.0 times and 1.7 times smaller than the controls (pplGal4/+) (Fig. 6A–D). Moreover, we observed a decrease of 2.8 times of EdU (5‐ethynyl‐2′‐deoxyuridine) incorporation in the CNS of larvae of second stage (Fig. 6E–G). These results clearly show that knockdown of ataxin‐2 directed by pplGal4 caused a decrease in the size of neuroblasts and affected the rate of neuron replication in the CNS, even though in this tissue the ataxin‐2 levels were not changed compared to control (Fig. 4C). This set of results indicates that larval growth reduction as a result of knockdown of ataxin‐2 in the fat body is due to decrease in the size and proliferation rate of the cells.

**Figure 7:**
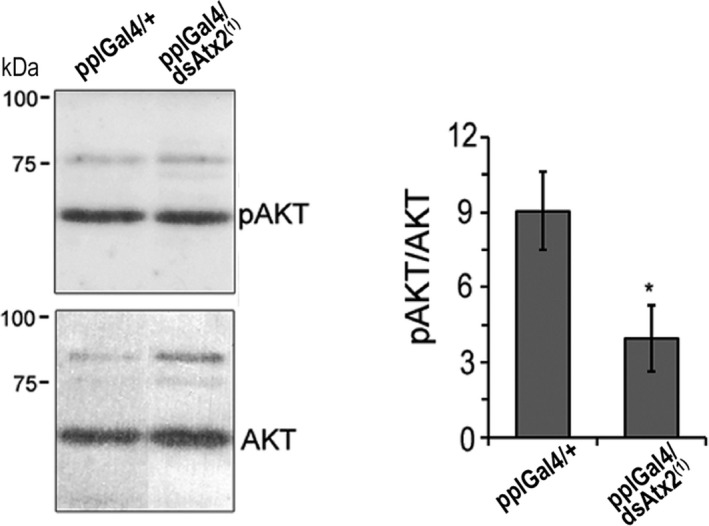
Silencing dAtx2 in the fat body reduces the levels of phosphorylated Akt. (A) Western blot of protein extracts from fat body of larvae expressing dsRNA against dAtx2 in the fat body (pplGal4/dsAtx2^(1)^) and control (pplGal4/+) probed with antibodies against pAkt (Ser 505) or Akt. The graph represents averages of the densitometric value ratio between pAkt and Akt, *n* = 3, *P < 0.05.

To assess whether the silencing of ataxin‐2 in the fat body interferes in the activity of the insulin/PI3K pathway, we measured the levels of phosphorylated AKT in relation to the unphosphorylated protein. We found that in the fat body of pplGal4/dsAtx2^(1)^ larvae the pAKT/AKT ratios were significantly reduced (~ 50%, *P < 0.01) compared to the control (pplGal4/+, Fig. 7), which indicates that the reduction in the levels of ataxin‐2 in fat body interferes with insulin/PI3K pathway upstream of AKT.

### Subcellular localization of ataxin‐2 in the fat body

**Figure 8:**
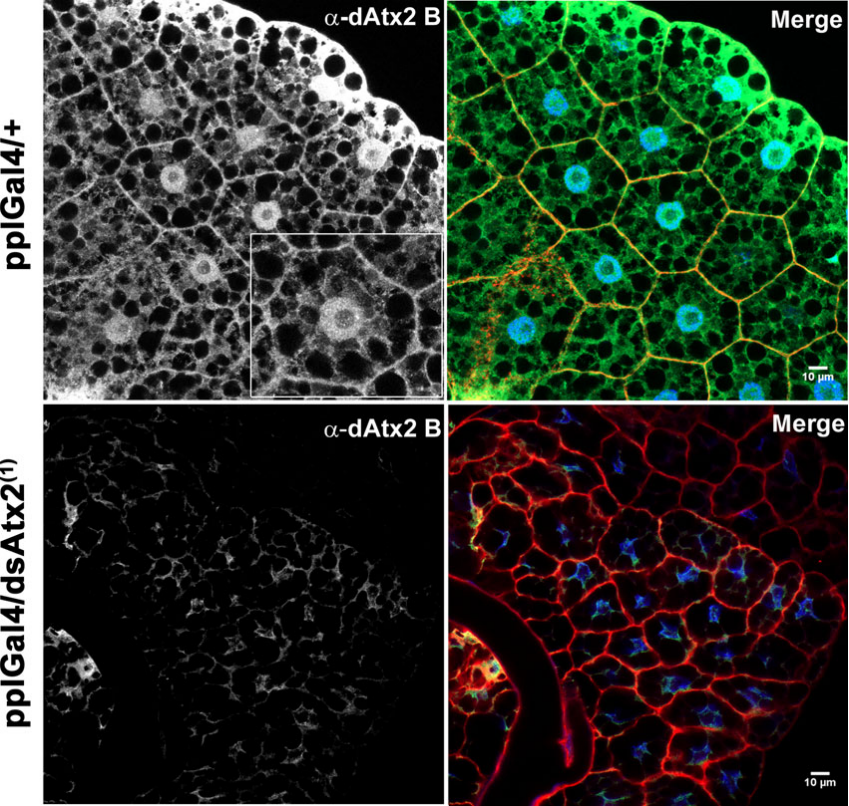
Ataxin‐2 B accumulates in the nuclei and in the cortex of fat body cells. Immunolocalization using α‐dAtx2 B and anti‐rabbit IgG conjugated to Alexa 488 (green). Actin filaments were stained with phalloidin conjugated to Alexa 594 (red) and nuclei with 4′,6‐diamidino‐2‐phenylindole, fluorescent stain that binds strongly to A‐T rich regions in DNA (blue). The images were captured using the Zeiss LSM 780 confocal microscope.

**Figure 9:**
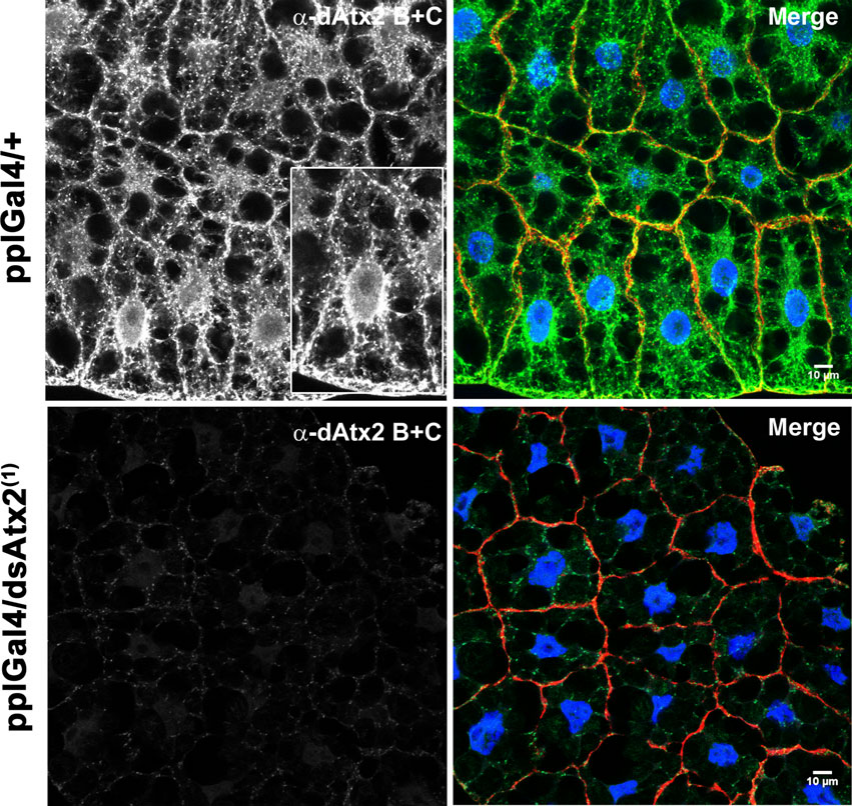
Immunolocalization of both ataxin‐2 B and C in the fat body cells of third instar larvae. Immunolabeling using α‐dAtx2 B + C and anti‐rabbit IgG conjugated to Alexa 488 (green). Actin filaments were stained with phalloidin conjugated to Alexa 594 (red) and nuclei with 4′,6‐diamidino‐2‐phenylindole, fluorescent stain that binds strongly to A‐T rich regions in DNA (blue). The images were captured using the Zeiss LSM 780 confocal microscope.

**Figure 10:**
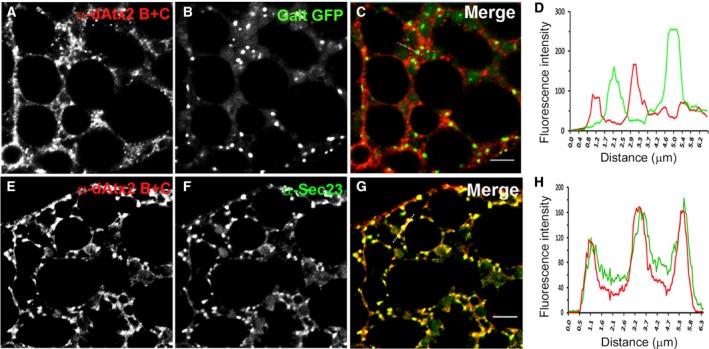
dAtx2 is excluded from the Golgi complex and accumulates in the ERESs in the fat body cells of third instar larvae. Immunolabeling of fat body of third instar larva. (A–C) α‐dAtx2 B + C and α‐rabbit IgG conjugated with Alexa 594 (red) of tissue expressing a resident protein of Golgi complex fused to GFP, (E–G) α‐dAtx2_C and α‐rabbit IgG conjugated with Alexa 594 of larvae expressing α‐dAtx2 B + C and α‐IgG of rabbit conjugated to Alexa 488 and α‐Sec23B and α‐IgY of chicken conjugated to Alexa 546. The graphs (D, H) show the profiles of the fluorescence intensity of ataxin‐2 and the markers as indicated by dashed line showed in (C, G), scale bar = 5 μm.

To gain further insight into the potential cellular roles of ataxin‐2 in the fat body, the subcellular distribution of these proteins was analyzed by comparison of the patterns of α‐dAtx2 B + C and α‐dAtx2 B labeling. The α‐dAtx2 B that recognizes a fragment specific of the B isoform (Fig. 1A) presented a fine granular labeling that was concentrated in the nuclei and in the cell cortex (Fig. 8). On the other hand the α‐dAtx2 B + C, that detects both dAtx2 isoforms, besides labeling the nuclei and cytoplasm cortex, accumulates in cytoplasmic foci with a diameter of ~ 1.4 μm (Fig. 9). Studies in mammalian cells have shown that ataxin‐2 may be located in the Golgi complex 30 and may cosediment with the rough endoplasmic reticulum (rER) membranes in the mouse brain 31. Thus, we sought to determine whether the large cytoplasmic foci labeled by α‐dAtx2 B + C were related to any of these organelles initially using a strain of flies expressing a marker to the Golgi apparatus. The results showed that the α‐dAtx2 B + C‐positive cytoplasmic foci are in the proximity but do not overlap the Golgi stacks, which are dispersed throughout the cytoplasm in Drosophila cells (Fig. 10A–D). Considering this pattern of α‐dAtx2 B + C label and the fact that in Drosophila the endoplasmic reticulum exit sites (ERESs) are almost always found in close association with Golgi stacks, forming the so called ‘tER‐Golgi units’ 32, we analyzed dAtx2 localization in relation to this particular region of the ER. Using an antibody against Sec23 as a marker of the ERESs, we demonstrate that α‐dAtx2 B + C‐labeled foci colocalized with α‐Sec23 (Fig. 10E–G) indicating that dAtx2‐C isoform is localized in this region of the rER.

## Discussion

By the analyses of RNA from S2R+ cells and animals at different developmental stages we confirmed the existence of the ataxin‐2 isoforms B and C of *Drosophila*, but not the predicted isoform A (Fig. 2). The dAtx2‐A differs from dAtx2‐C mRNA structure only by the retention of the last intron, which creates a premature stop codon (Fig. 1A) indicating that the dAtx2‐A cDNA probably represents an incompletely processed dAtx2‐C mRNA precursor. The protein encoded by dAtx2‐C represents a 61 aa truncated N‐terminal form of dAtx2‐B. The polyclonal antibodies generated against a fragment common to both isoforms C and B, and the antibody specific for isoform B, detected bands of ~ 150 kDa (Fig. 2E). The SDS/PAGE mobility pattern of the polypeptides detected by both antibodies agrees with previously reported data obtained with an antiserum raised against a synthetic polypeptide common to both dAtx2 isoforms, and whose specificity was demonstrated by the use of mutant lines 33. The dAtx2 molecular mass estimated in SDS/PAGE differs from the masses of the polypeptides deduced from the dAtx2‐B and dAtx2‐C transcripts, which are 117.6 and 111.4 kDa, respectively. Such size discrepancies were also observed in mammalian ataxin‐2 and might be due to post‐translational changes in these proteins, such as phosphorylation 34.

The analysis of *dAtx2* expression at the mRNA and protein levels indicates that the expression of both dAtx2‐B and ‐C isoforms are differentially regulated. While the dAtx2‐B mRNA levels are relatively low throughout development, dAtx2‐C amounts increase significantly from the L3 to adulthood (Fig. 2B). However, the dAtx2‐B protein levels are significantly higher in embryos and L1 larvae than in animals at later developmental stages (Fig. 3A), suggesting that expression of this isoform is controlled at post‐transcriptional levels. Moreover, while the levels of dAtx2‐B protein are reduced after L1, the intensity of α‐dAtx2 B + C signal that recognizes both dAtx2‐B and ‐C isoforms is maintained high (Fig. 3A), which is consistent with the induction of the dAtx2‐C mRNA expression at the end of the larval period (Fig. 2C). Based on these observations, and the fact that *dAtx2* mutants die in the second larval instar 33 we propose that the dAtx2‐C isoform functions are required for larval–pupal stage transition. Corroborating a previous report 33, the levels of ataxin‐2 detected with both antibodies in adult ovaries were higher than in the other analyzed tissues (Fig. 3B) indicating that both isoforms B and C could be maternally supplied, thus providing ataxin‐2 functions at the beginning of development.

Also consistent with the evidence that *dAtx2* function is essential for development 33 we observed that RNA‐interference reduction of ataxin‐2 in larval fat body arrests development at prepupal stage. The metamorphosis is not complete and the L3 larvae expressing reduced levels of ataxin‐2 in the fat body are significantly smaller than the controls (Fig. 4) suggesting that the function of ataxin‐2 in this tissue is necessary for its role in coordinating growth and metamorphosis 29. It has been demonstrated that suppression of insulin/PI3K pathway by overexpression of Δp60 or PTEN, predominantly in the fat body, results in delayed development and reduced size of the individual due to the smaller number and size of the cells 28. In agreement with the hypothesis that the silencing of *dAtx2* interferes with insulin/PI3K pathway, our data show a positive correlation between the levels of ataxin‐2 and pAkt in the fat body (Fig. 7). Interestingly, ataxin‐2 knockout mice present a reduction in insulin receptor levels in liver and cerebellum, although the amounts of mRNA are increased, which indicates a post‐transcriptional role of ataxin‐2 in the control of this receptor expression 23. Furthermore, studies in mammals indicate that lower levels of ataxin‐2 affects the traffic of endocytic epidermal growth (EGFR) receptor, suggesting a possible role of this protein in the receptor recycling 22. Moreover, there are reports that ataxin‐2 interacts with components of the endocytic machinery such as endophilin A1, Scr, and CIN85 22, 35, 36. This interaction occurs through the SH3 motif present in this class of proteins that has a high affinity to proline‐rich domains, such as ATXN2 35. Interestingly, ataxin‐2 of *Drosophila* has at its carboxy‐terminal portion, a high concentration of proline and so may potentially bind to proteins involved in the endocytosis process. The accumulation of dAtx2, particularly the isoform B (Fig. 8), in the cell cortex of the larval fat body is compatible with its involvement in these protein complexes. Accumulation of ataxin‐2 in the cell cortex was earlier shown in mammal cells 30.

Indeed, multiple cellular processes in the fat body might require ataxin‐2 function to regulate development. A nuclear role must be considered since dAtx2‐B label accumulates in the nuclei as well as in the cell cortex (Fig. 8). In mammalian cells it has been proposed that ATXN2 might modulate transcription, since it was shown to relocalize to the nucleus and control its own expression 24. Moreover Lsm domains in ATXN2 are generally found in proteins that function in mRNA splicing 37 and the Ataxin‐2‐binding protein 1 (A2BP1/FOX1) is a well‐established splicing modulator in other metazoans 38, 39. However, different than dAtx2‐B, in fat body cells the dAtx2‐C isoform seems to be concentrated in the ERESs (Fig. 10I–L). Studies in human cells showed that deletion of portions of ataxin‐2 containing the ER exit and clathrin‐mediated signals results in alteration of the subcellular localization of the protein, suggesting that these signals are functional 30. From yeast to human, it is estimated that approximately one‐third of the proteome is synthesized in the ER and transits to membrane compartments such as the plasma membrane or undergoes secretion. Thus, an impact on ERES organization in fat body by the reduction of ataxin‐2 could have a direct relationship with the endocrine role that this tissue has on the growth and metamorphosis in Drosophila 29, 40. This could explain the arrest of development observed when ataxin‐2 levels are reduced in the fat body (Figs 4, 5, 6) as well as the reduction in PI3K signaling in this tissue (Fig. 7). Ataxin‐2 is a RNA‐binding protein and a number of recent *in vivo* studies support its role in translation regulation 17, 18, 19, 20, 21. Interestingly, previous studies indicate that normal ERES formation in Drosophila oocytes is locally regulated by RNA‐protein complex containing the translation/localization factors Tral, Me31B, and Cup 41. Based on these observations we raise the possibility that ataxin‐2 may be required for normal ERES formation in Drosophila fat body and so could be necessary for efficient protein trafficking in the cell. This putative cellular function for dAtx2‐C should be further investigated, especially in CNS, considering also that interferences in exocytose pathway may induce ER stress which has been proposed as an important driver of neurodegenerative diseases, including ALS 42.

## Materials and methods

### *Drosophila* lines

The fly strains used were: UAS‐Atx2^(1)^ RNAi (GD34956) and UAS‐Atx2^(2)^ RNAi (GD34955) obtained from Vienna *Drosophila* RNAi Center stock collection (VDRC); pplGal4, P{ppl‐GAL4.P}3, gently provided by Dr. Alex Gould, MRC National Institute for Medical Research, England; P{UASp‐GFP.Golgi}1, obtained from Bloomington Stock Center, Canton S, and yw obtained from Dr. M. Jacobs‐Lorena, Johns Hopkins Bloomberg School of Public Health, USA.

### Antibodies

Rabbits were injected with His‐tagged fragments of dAtx2 expressed in *E. coli*. The fragments consisted of 60 amino acids specific of dAtx2‐B (positions 1–61), and 104 amino acids polypeptide, common to the predicted dAtx2 isoforms (amino acid positions 271–375 in dAtx2‐B). Both antibodies, dAtx2 B (against the 60 aa) and dAtx2 B + C (against the 104 aa), were immunopurified by incubation with a nitrocellulose membrane with previously bound fusion fragments. After elution with triethylamine, followed by neutralization with 1 m Tris‐Cl, pH 8.0, the antibodies were microdialyzed against TBS (0.15 m NaCl; 0.1 m Tris, pH 8.0). α‐Sec23B (ab37739) chicken polyclonal Abcam (Cambridge, UK), anti‐α‐tubulin (#T5168) mouse monoclonal Sigma (St. Louis, MO, USA), α‐Akt (#9272) rabbit polyclonal Cell Signaling (Beverley, MA, USA), α‐Akt Ser 505 (#4054) rabbit polyclonal Cell Signaling, α‐dpn rat polyclonal, gently provided by Prof. Dr. Cheng‐Yu Lee, Howard Hughes Medical Institute, University of Oregon. All efforts were made to minimize the number of animals used to raise the dAtx2 B and dAtx2 B + C antibodies and to avoid any unnecessary suffering conformed to ethical guidelines presented by Public Health Service, National Institutes of Health Publication 86‐23.

### RNA extraction, northern blot and RT‐PCR analysis

*Drosophila* total RNA was extracted using Trizol reagent (Invitrogen, Carlsbad, CA, USA) according to the manufacturer's instructions. Northern blot hybridization was performed using standard methods. For RT‐PCR, the polyA+ RNA samples were treated with DNase I (Promega, Fitchburg, WI, USA) and converted to cDNA with SuperScript III (Invitrogen). PCR reactions were performed using Taq DNA polimerase (Amersham Biosciences, Piscataway, NJ, USA) and the primers: dAtx2_F1 (5′ atcgctacagtacgcaggg 3′), dAtx2_F2 (5′ attgaaaacccgcgacaac 3′), dAtx2_1‐2R (5′ ttggacttaatgcacgccgg 3′), dAtx2_3F (5′ gccagtacgcctacagcag 3′), dAtx2_3R (5′ ggaattgttgctggtgtggt 3′), Rp49_F (5′ catccgcccagcatacagg 3′), Rp49_R (5′ ccgttggggttggtgagg 3′). dAtx2 primers were designed based on sequences available in the Flybase. Their positions in the mRNA models are shown in Fig. 1.

For quantitative PCR of isoform B and C we used the SYBR Green PCR Master Mix (PE Applied Biosystems, Waltham, MA, USA), according to the manufacturer's protocol, and the following primers: Atx2‐B_F (5′ accgaaagcgccacaaagac 3′), Atx2‐C_F (5′ acaatagcaagcggaaaaccc 3′), Atx2‐B/C_R (5′ tccgttcccgcctcctgc 3′), RpL32A F (5′ gaccatccgcccagcatac 3′), RpL32A_R (5′ cgcactctgttgtcgatacc 3′). Reactions without template were run in parallel for all plates to verify purity of measurements within each experiment. Each run was completed with a melting curve analysis to confirm the specificity of the amplification and to confirm absence of primer dimers. The mean threshold cycle of a mixture of equal amounts of fat body, ovary, and carcass RNA was used as a reference sample. The relative mRNA expression levels of target transcripts and the RpL32A gene (housekeeping) were quantified using a Gene Amp^®^ 7500 Sequence Detection System (PE Applied Biosystems). The qRT‐PCR analyses were repeated three times and data were analyzed statistically by one‐way analysis of variance (ANOVA) and by the Tukey multiple‐comparison test (graphpad prism, version 5.0; La Jolla, CA, USA).

### Protein extraction and immuno blot

Tissues were dissected in cold PBS containing Bestatin; Pepstatin; 2 μg·mL^−1^ Aprotinin; 1 mm Benzamidin, 1 mm PMSF. Dissected tissues and whole animals at different stages were homogeneized in sample buffer 2× (18% Glycerin; 125 mm Tris‐HCl pH 6.8; 10% β‐mercaptoethanol; 0.01% bromophenol blue). SDS/PAGE and western blots were performed using standard methods. The following primary antibodies were used: α‐dATX2 B (1 : 500), α‐dATX2 B + C (1 : 500), and anti‐α‐tubulin mAb 1 : 1000 (Sigma‐Aldrich). Bound primary antibodies were detected by chemiluminescence (Amershan ECL kit, GE Healthcare UK Limited, Little Chalfont, UK). For measurements of the phosphorylated AKT in relation to the unphosphorylated protein, the same membranes were first probed with α‐Akt Ser 505 (pAKT),followed by α‐Akt probing, after membrane regeneration.

### Microscopy, imunofluorescence, and area measurement

The CNS, fat body, and salivary glands were fixed and permeabilized in solution containing 4% formaldehyde; 0.1 m PIPES, pH 6.9; 0.3% Triton X‐100; 20 mm EGTA pH 8.0, and 1 mm MgSO4, for 20 min at room temperature and washed twice for 30 min in PBST (PBS pH 7.4 and 0.3% Triton X‐100). Blocking was carried out in PBSBT (PBST, 1% BSA, goat serum, and 0.1% glycine 0.01 m) for at least 2 h. For deadpan detection tissue was incubated with α‐dpn (1 : 1) for 3 h at room temperature. For ataxin‐2, the incubation with the antibodies dAtx2 B + C and dAtx2 B(2 μg·mL^−1^) was performed overnight at 4 °C. For ataxin‐2 and sec‐23 colabeling, the α‐sec23B (1 : 300) was coincubated with the secondary antibody for α‐dAtx2 B + C. The preparations were washed for 30 min in 2× PBSBT followed by incubation at 4 °C overnight with the adequate secondary antibody; α‐chicken Alexa 594 (1 : 300) for α‐sec23B, α‐rat Texas Red (1 : 300) for α‐dpn, and α‐rabbit Alexa 488 or 594 (1 : 300) for ataxin‐2 detection. Actin filaments were detected with phalloidin conjugated to Alexa Fluor 488 or Alexa Fluor 594 (1 : 200; Molecular Probes, Waltham, MA, USA) and nuclei were stained with 4′,6‐diamidino‐2‐phenylindole, fluorescent stain that binds strongly to A‐T rich regions in DNA (1 μg·mL^−1^) for 30 min at room temperature. The tissues were washed two times in PBS for 30 min, and mounted in antifade (ProlongGold; Molecular Probes) solution. The samples were examined using Leica TCS SP5 confocal microscope or Zeiss LSM 780 Confocal Microscope. Measurements of the cells/nuclei and cerebral lobes areas were performed using imagej MacBioPhotonics (http://imagej.net/mbf/).

### Proliferation assay by incorporation of EdU

The CNS second‐stage larvae (48 h after egg deposition) were dissected and incubated for 1 h in 10 μm EdU (5‐ethynyl‐2′‐deoxyuridine) diluted in PBS. Soon after, the tissues were fixed in 3.7% formaldehyde for 10 min and washed quickly 2× in PBS with 3% BSA. Permeabilization was performed with 0.5% Triton X‐100 for 15 min, followed by 2× wash in PBS with 3% BSA. The Alexa Fluor 594 was covalently linked to EdU according to the manufacturer's instructions (Click‐iT EdU Imaging Kit; Invitrogen). Tissues were mounted in antifade solution (ProlongGold; Molecular Probes) and examined under Leica TCS SP5 Confocal Microscope. The quantification of fluorescence intensities and area of CNS were performed using imagej MacBioPhotonics (http://imagej.net/mbf/).

## Author contributions

MLPL and VV planned the experiments; MCBV, DCP, and PFG performed the experiments; MCBV, DCP, PFG, and MLPL analyzed the data; MCBV and MLPL prepared the first draft of the manuscript; MLPL wrote the paper; MLPL, VV, MCBV, and PFG reviewed the paper.
